# Recent progress in combination therapy of oncolytic vaccinia virus

**DOI:** 10.3389/fimmu.2024.1272351

**Published:** 2024-03-13

**Authors:** Seyedeh Nasim Mirbahari, Miles Da Silva, Abril Ixchel Muñoz Zúñiga, Nika Kooshki Zamani, Gabriel St-Laurent, Mehdi Totonchi, Taha Azad

**Affiliations:** ^1^ Faculty of Sciences and Advanced Technologies in Biology, University of Science and Culture, Academic Center for Education, Culture and Research (ACECR), Tehran, Iran; ^2^ Department of Genetics, Reproductive Biomedicine Research Center, Royan Institute for Reproductive Biomedicine, Academic Center for Education, Culture and Research (ACECR), Tehran, Iran; ^3^ Department of Microbiology and Immunology, University of British Colombia, Vancouver, BC, Canada; ^4^ Ottawa Hospital Research Institute, The Ottawa Hospital, Ottawa, ON, Canada; ^5^ Department of Microbiology and Infectious Diseases, Université de Sherbrooke, Sherbrooke, QC, Canada; ^6^ Centre de Recherche du CHUS, Sherbrooke, QC, Canada

**Keywords:** oncolytic virus, oncolytic vaccinia virus, combination therapy, cancer therapy, immunotherapy

## Abstract

In recent years, oncolytic viruses have emerged as promising agents for treating various cancers. An oncolytic virus is a non-pathogenic virus that, due to genetic manipulation, tends to replicate in and cause lysis of cancerous cells while leaving healthy cells unaffected. Among these viruses, vaccinia virus is an attractive platform for use as an oncolytic platform due to its 190 Kb genome with a high capacity for encoding therapeutic payloads. Combining oncolytic VV therapy with other conventional cancer treatments has been shown to be synergistic and more effective than monotherapies. Additionally, OVV can be used as a vector to deliver therapeutic payloads, alone or in combination with other treatments, to increase overall efficacy. Here, we present a comprehensive analysis of preclinical and clinical studies that have evaluated the efficacy of oncolytic vaccinia viruses in cancer immunotherapy. We discuss the outcomes of these studies, including tumor regression rates, overall survival benefits, and long-term responses. Moreover, we provide insights into the challenges and limitations associated with oncolytic vaccinia virus- based therapies, including immune evasion mechanisms, potential toxicities, and the development of resistance.

## Introduction

In recent years, innovative approaches have emerged for developing new biological therapies for cancer treatment, including CAR-T cell therapy and oncolytic viruses (OV), either alone or in combination with traditional treatments. OVs have shown remarkable efficacy against different types of cancers for over three decades (1).

Oncolytic viruses execute their anticancer activity through a multifaceted interplay of intricate mechanisms designed to selectively target and eliminate cancer cells. Upon administration, OVs specifically recognize and infect tumor cells. This specificity is achieved through a variety of genetic manipulations that exploit cancer cells inherent vulnerabilities. One primary mechanism involves the direct lysis of infected cancer cells, where viral replication within the tumor leads to cell lysis, causing the release of progeny viruses and intracellular contents (2, 3). Concurrently, the viral infection triggers immunogenic cell death, promoting the release of danger signals and facilitating the activation of antitumor immune responses. The immune system, now recruited and activated, recognizes and eliminates both infected and non-infected neighboring cancer cells, creating a systemic antitumor immune response (4). Furthermore, oncolytic viruses can induce a cascade of immunomodulatory effects within the tumor microenvironment, enhancing the recruitment and activation of immune cells, such as cytotoxic T lymphocytes and natural killer cells (5). OVs can also disrupt tumor vasculature, impeding blood supply to the tumor and contributing to the antitumor effect (6). In addition of its primary function, OVs can lead to expression of therapeutic transgene in the tumor microenvironment (TME) following infection of tumor cells. This feature increased the oncolytic potential and therefore is important for future combination of oncolytic therapy and immunotherapy. These mechanisms allow scientists to hijack the immune system to create OV-elicited antitumor immunity. Relative contributions of each mechanism depend on the nature and type of cancer cell, the characteristics of the viral vector, and interactions between the virus, tumor microenvironment and host immune system. These multifaceted mechanisms collectively contribute to the selective elimination of cancer cells by oncolytic viruses, presenting a promising avenue for innovative and targeted cancer immunotherapies.

OV therapy implicates different mechanisms, like interactions between tumor cells, viruses, and the immune system. The anti-tumor activity of the OVs can be identified in two categories, the killing of tumor cells, which is determined by the expression of the receptor in the cell surface, and the host cell’s anti-viral response. The other mechanism benefits the generation of a proinflammatory tumor microenvironment, boosting systematic anti-tumor immunity (7).

Several approaches have been developed to engineer viruses to make them oncolytic, including the downregulation of viral genes necessary for replication in healthy cells, the use of tissue or tumor-specific promoters. It has been reported that OVs replicate more efficiently within tumor cells due to a tumor cell’s deficiency in antiviral type I interferon signaling (8). Overall, oncolytic vaccinia virus (9) can infect healthy and tumors cells, but genetic modifications could make them selective for replication in tumor cells. Non-invasive imaging techniques, such as reporter transgenes, allow the tracking and tumor specificity evaluation of OVs (10). OVV has shown promising results in preclinical, phase-1, -2 and -3 clinical trials, and has a long history of safety (11–13). OVVs can infect tumor cells and replicate constantly, therefore causing oncolysis. By infecting tumor cells, tumor-associates molecular patterns are exposed, including antigens, DAMPs, PAMPs, and cytokines. These elements thereafter activate the immune responses in the tumor microenvironment. Furthermore, these patterns stimulate innate and adaptative immunity (14). However, treatment response may vary depending on the differential baseline immunological profile of each patient (15). **Table 1** lists the OVVs currently in clinical trials, their specific genetic modifications, and any therapeutic combinations. The vaccinia virus (9) is an enveloped virus with a linear dsDNA genome of approximately 190 Kb containing approximately 250 protein-coding genes. It was the first safe and effective human vaccine, ultimately eradicating smallpox (13). Since VV replicates entirely in the cytoplasm of infected cells, there is no concern about the possibility of intra-nuclear mutagenesis. A wide range of cells are considered to be hosts of vaccinia. The virus enters host cells by an endocytic process through the cell membrane (35, 36). VV can express up to 50 kb of external DNA and can express multiple therapeutic genes simultaneously. Vaccinia has a rapid and lytic replication cycle that triggers a robust immune response and inflammation in the host (37). The host immune system easily controls the infection, however, in case of uncontrolled virus growth, antiviral treatments such as ST-246 (tecovirimat) and cidofovir exist (38, 39). During VV maturation, newly assembled immature virions are enveloped in a membrane to form infectious intracellular mature virions (IMV), which are further enveloped in a double membrane layer to form intracellular enveloped viruses (IEVs) (40). The outermost IEV membrane layer then fuses with the cytoplasmic membrane to release the virion from the host cell. Tecovirimat inhibits the VP37 protein, which is needed for IMV membrane envelopment to form IEV. The availability of tecovirimat supports the development of OVV therapies by providing an option to treat severe adverse effects of OVVs or reduce adverse effects of higher dose OVV use (41).

**Table 1:** List of OVVs (alone or in combination with other treatments) in different phases of clinical trial.

Vaccinia Virus	Gene Modification	Combination	Phase of clinical trial	Conditions	NCT Number	Ref.
JX-929 (vvDD)	VGF and TK deletion	Alone	Phase I	Breast, pancreas and colon, melanoma cancer	–	(16, 17)
JX-594 (Pexa Vec)	TK-deletion plus GM-CSF and lac Z	Sorafenib	Phase III	Hepatocellular Carcinoma (HCC)	NCT02562755	(18)
BT-001	Encoding Treg-depleting human recombinant anti-CTLA4 antibody and GM-CSF	Pembrolizumab	Phase I, II	Metastatic Cancer|Soft Tissue Sarcoma|Merkel Cell Carcinoma|Melanoma|Triple Negative Breast Cancer|Non Small Cell Lung Cancer	NCT04725331	(19)
KM1	–	Chemotherapy	Phase I	Ovarian Cancer	NCT05684731	No publications available
GL-ONC1	TK, hemagglutinin and F14.5L deletion	Alone	Phase I	Advanced Cancers (Solid Tumors)	NCT00794131	(20)
GL-ONC1	TK, hemagglutinin and F14.5L deletion	Alone, or in combination with chemotherapy with or without bevacizumab	Phase I, II	Ovarian Cancer|Peritoneal Carcinomatosis|Fallopian Tube Cancer	NCT02759588	(21)
T601	Tyrosine kinase (TK), ribonucleotide and reductase (RR) deetion plus yeast-originated bifunctional cytosine deaminase and Uracilphosphoribosyltransferase gene (FCU1)	5-FC	Phase I, II	Advanced Malignant Solid Tumors	NCT04226066	No publications available
ASP9801	Encoding IL-7 and IL-12	Pembrolizumab	Phase I	Metastatic Cancer|Solid Tumors|Advanced Cancer	NCT03954067	(22)
RGV004	Encoding anti-CD19/CD3 bispecific antibody	Alone	Phase I	Relapsed or Refractory B-cell Lymphoma	NCT04887025	–
JX-594 (Pexa Vec)	Recombinant vaccinia virus (TK-deletion plus GM-CSF)	Best Supportive Care	Phase II	Hepatocellular Carcinoma|Liver Cancer|HCC	NCT01387555	(23)
JX-594 (Pexa Vec)	Recombinant vaccinia virus (TK-deletion plus GM-CSF)	Alone	Phase I, II	Melanoma	NCT00429312	(11)
JX-594 (Pexa Vec)	Recombinant vaccinia virus (TK-deletion plus GM-CSF)	Alone	Phase II	Carcinoma, Hepatocellular	NCT00554372	(12, 24)
JX-594 (Pexa Vec)	Recombinant vaccinia virus (TK-deletion plus GM-CSF)	Durvalumab Tremelimumab|	Phase I, II	Colorectal Cancer|Colorectal Carcinoma|Colorectal Adenocarcinoma|Refractory Cancer|Colorectal Neoplasms	NCT03206073	(25, 26)
GL-ONC1	TK, hemagglutinin and F14.5L deletion	Alone	Phase I, II	Peritoneal Carcinomatosis	NCT01443260	(20)
VV-GMCSF-Lact	expressing exogenous proteins: the antitumor protein lactaptin and (GM-CSF)	Alone	Phase I	Oncolytic Virotherapy	NCT05376527	(27)
JX-594 (Pexa Vec)	Recombinant vaccinia virus z	Irinotecan	Phase I, II	Colorectal Carcinoma|CRC	NCT01394939	(28)
Olvimulogene nanivacirepvec (GL-ONC1)	TK, hemagglutinin and F14.5L deletion	Platinum chemotherapy: carboplatin (preferred) or cisplatin Non-platinum chemotherapy: Physician’s Choice of gemcitabine, taxane (paclitaxel, docetaxel or nab-paclitaxel) or pegylated liposomal doxorubicin Bevacizumab (or biosimilar	Phase III	Platinum-resistant Ovarian Cancer|Platinum-refractory Ovarian Cancer|Fallopian Tube Cancer|Primary Peritoneal Cancer|High-grade Serous Ovarian Cancer|Endometrioid Ovarian Cancer|Ovarian Clear Cell Carcinoma	NCT05281471	(29)
JX-594 (Pexa Vec)	Recombinant vaccinia virus (TK-deletion plus GM-CSF)	Alone	Phase I	Melanoma|Lung Cancer|Renal Cell Carcinoma|Squamous Cell Carcinoma of the Head and Neck	NCT00625456	(30)
JX-594 (Pexa Vec)	Recombinant vaccinia virus (TK-deletion plus GM-CSF	Alone	Phase I	Neoplasms, Liver	NCT00629759	(31)
GL-ONC1	TK, hemagglutinin and F14.5L deletion	Alone	Phase I	Cancer of Head and Neck	NCT01584284	(32)
JX-594 (Pexa Vec)	Recombinant vaccinia virus TK-deletion plus GM-CSF	Alone	Phase II	Ovarian Cancer	NCT02017678	–
JX-594 (Pexa Vec)	Recombinant vaccinia virus (TK-deletion plus GM-CSF	Alone	Phase I	Neuroblastoma|Rhabdomyosarcoma|Lymphoma|Wilm’s Tumor|Ewing’s Sarcoma	NCT01169584	(30)
TBio-6517	Plus anti-CTLA-4 antibody, FLT3 ligand (FLT3L), Membrane- bound IL-12 (p35 subunit)	Pembrolizumab	Phase I, II	Solid Tumor|Microsatellite Stable Colorectal Cancer|HPV Positive Oropharyngeal Squamous Cell Carcinoma|Cervical Cancer|Melanoma (33) s|Cutaneous Squamous Cell Carcinoma|Mesothelioma|Renal Cell Carcinoma|Oropharynx Cancer	NCT04301011	–
Recombinant human IL-21 oncolytic vaccinia virus injection	deletion of TK gene (TTVΔTK) and armed rationally with IL-21	Alone	Phase I	Advanced Solid Tumors	NCT05914376	(34)

**Figure 1:**
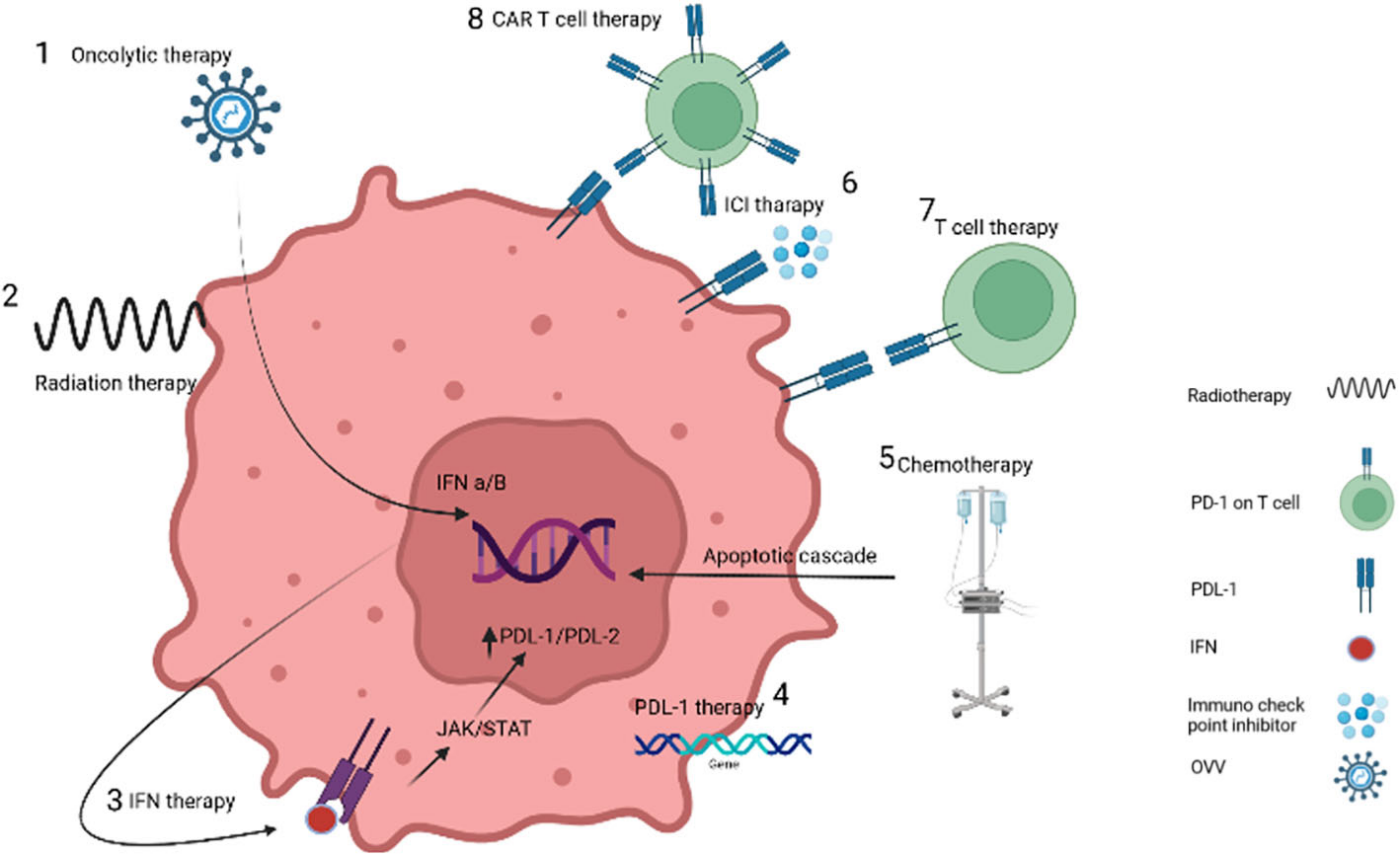
Targeting cancer cells by different treatment methods. 1. OVVs increase the expression of interferon by entering cancer cells. 2. Radiotherapy causes damage to the DNA of the tumor cell, while it does not damage the DNA of the vaccinia virus due to the difference in the time of injection. 3. Interferon therapy recruit immune cells. 4. PDL-1 protein injection or gene insertion increases the response to immunotherapy methods. 5. Chemotherapy destroys cancer cells by causing inflammation. 6. Blockage of immunochecking inhibitors such as anti-PD-1 causes the recognition of receptors by T-cells. 7 and 8 T-cells and CAR-T cells also destroy cancer cells by binding receptors.

Recent advancements in molecular biology have made VV an attractive candidate for engineering as an oncolytic agent (40). With the genetic diversity of tumor types and their resistance to routine drugs, monotherapies have limited success in treating many cancer types. Combination methods can be more effective and have reinforcing results on treatment, but finding these combinations can be challenging. **Figure 1** illustrates the different types of cancer treatments that have demonstrated synergistic effects with OVV therapy. This review will summarize recent applications of OVV treatment combinations and discuss their potential for improving the response to treatment.

## OVVs as a next generation of immunotherapeutic to remodel TME

### Immune checkpoint inhibitor antibody therapy

Immune checkpoint molecules are proteins found on the surface of cells that help regulate the immune system’s response to foreign invaders, such as cancer cells. These molecules act as “checkpoints’’, either activating or inhibiting the immune response. Some cancer cells can evade the immune system by exploiting these inhibitory checkpoint molecules, allowing them to avoid detection by lymphocytes and thus grow and spread. Immune checkpoint inhibitors (ICIs) are a class of drugs that target these checkpoint molecules to restore the immune system’s ability to attack cancer cells. Currently, ICIs are used as a treatment for certain types of cancer, such as melanoma and lung cancer. Several inhibitory checkpoint molecules have been identified, including CTLA-4, PD-1, CD80/86, MHC II, Galectin-3, FGL1, CD112, CD155, HVEM, and Ceacam-1, known as interferon (IFN) signaling-stimulated genes (ISGs) (42–45). Even though ICIs have shown promise in the treatment of different types of cancer, monotherapy rarely offers long-term benefits for most patients. Despite the capacity of CAR-T cell therapy to enhance T-cells to target cancer cells which is efficacious in treating hematologic malignancies, its ability to treat solid tumors is limited (14). Therefore, combining OVs with ICIs is a common way to increase treatment effectiveness, as both therapies work to relieve the tumor’s immunosuppressive environment. Additionally, OV infection triggers an anticancer immune response, increasing therapy efficacy (46–51). Various ICIs have been developed, with each targeting different checkpoint molecules. Examples include CTLA-4 inhibitors like ipilimumab, PD-1 inhibitors such as nivolumab and pembrolizumab, and LAG-3 inhibitors like MEDI3039, which block the activity of the respective proteins found on the surface of T-cells (52).

Programmed death receptor (PD-1), also known as CD279, is a type 1 transmembrane protein encoded by the PDCD1 gene of the CD28 immunoglobulin superfamily (53). PD-1 is 288 amino acids long and has a single Ig variable-type (IgV) extracellular domain, a transmembrane domain, and a cytoplasmic domain (54). It is mainly expressed in activated CD4+ T-cells, CD8+ T-cells, natural killer T-cells, B-cells, macrophages, dendritic cells (DCs) and monocytes. Its expression is induced by the T- or B-cell receptor pathway and enhanced by the stimulation of tumor necrosis factor (55). Naive T and B-cells barely express PD-1 (56). The biological functions of PD-1 rely on two ligands: PD-L1 (also known as B7-H1 or CD274) and PD-L2 (also known as B7-H2 or CD273). PD-L1 is constitutively expressed on T-cells, B-cells, DCs, cancer cells, macrophages and others, and is further upregulated by activated pro-inflammatory cytokines (57). PD-1/PD-L1 interaction is mainly responsible for the immune escape of cancers. When PD-L1 binds to PD-1, two tyrosines of the immune-receptor tyrosine-based switch motif (ITSM) region of PD-1’s intracellular domain is phosphorylated. These phosphorylated tyrosines then phosphorylate the B-cell receptor (BCR), and SHP-2, a phosphatase, binds to the C-terminal of PD-1. Subsequently, phosphorylated SHP-2 dephosphorylates the BCR, leading to impaired Ca2+ ion generation and long-term growth arrest (58). In normal cells, PD-1/PD-L1 binding prevents the overstimulation of T-cells and maintains immune tolerance to antigens, preventing the development of autoimmune diseases. In cancer cells, the binding of PD-L1 to PD-1 generates negative signals, which induce T-cell apoptosis and reduce immunocompetence, aiding cancer cells in avoiding recognition by the immune system. Additionally, activation of the PD-1/PD-L1 pathway negatively affects the differentiation of effector T-cells (59), memory T-cells (60), regulatory T-cells (Treg), and exhausted T-cells (61), greatly reducing the effect of T-cells on tumor cells (60). Interestingly, PD-L1 carried in exosomes can be transported to remote regions of the body via the circulatory system. This immigrated PD-L1 inhibits T-cell activity remotely before reaching metastatic lesions (62). Blocking the binding of PD-L1 to PD-1 inhibits these negative consequences, thereby preserving T-cell function and their ability to kill cancer cells.

A present-day study demonstrates the strengthening of systemic anti-tumor effects in various preclinical tumor models, with a vaccinia virus that coexpresses a PD-L1 inhibitor and GM-CSF (VV-iPDL1/GM). This proposal effectively suppressed PD-L1 expression in tumor and immune cells which therefore increased infiltration and activation of tumor-specific T-cells. These react against neoantigen epitopes derived from mutations, which results in a successful rejection of both virus-injected and distant tumors (63).

Today, ICI immunotherapy shows promise in treating various solid tumor cancers and hematological malignancies, with durable responses and long-term survival benefits (64, 65). Blocking ICIs can be combined with OVV therapy using two strategies: anti-ICI antibody genes can be inserted into the OVV genome, which is then categorized as an immuno-oncolytic virus, or patients can receive an injection of anti-ICIs antibodies before or after OVV therapy. Immuno-oncolytic viruses will be discussed further in the next section. **Table 2** displays different ICI with OVV combination therapy studies, some applying the gene insertion technique while others utilize antibody injections. In a 2022 study, a regimen of 4 different therapies was designed, which included an OVV known as GLV-1h68, melphalan, TNFα, and intra-peritoneal injection of recombinant PD-1 protein, followed by completion with radiotherapy. The combination of these therapies recruited CD4+ and CD8+ T-cells and significantly altered the TME (69). In another study, OVV mpJX-594 (mpJX) was intravenously injected alone or together with doses of PD-1 into functional and metastatic pancreatic neuroendocrine tumors (PanNETs). Simultaneous injection of PD-1 with mpJX had a synergistic effect on the secretion of NK, CD8+ T-cell, leading to apoptosis and inhibition of the proliferation of pancreatic tumor cells. This therapeutic combination increased survival and anti-metastatic activity in liver metastasis model mice (76).

**Table 2:** OVV in combination with ICIs in different studies.

OVV	Condition	ICIs (Combined treatment)	Ref.
ΔTK-ARMED-VACV	Pancrearic and hepatic cell lines	Coding sequence of *anti-PD-1* and *anti-4-1BB*	(66)
hIL-7/mIL-12- VV	Melanoma, colon, lung, prostate adenocarcinoma, breast renal adenocarcinoma, melanoma, head and neck, glioblastoma neuroblastoma, hepatocellular carcinoma, esophageal, colorectal, gastric, bladder, kidney, ovarian, cervical and breast cancer	Anti–programmed cell death-1 (PD-1) or anti–cytotoxic T lymphocyte antigen 4 (CTLA4) antibody	(22)
VV-iPDL1/GM	Osteosarcoma, adenocarcinoma, melanoma, murine lymphoma	Coding sequence of *anti-PD-1* and *GM-CSF*	(63)
vvDD-IL15-Rα,	Mice bearing colon and ovarian cancer	Anti-PD-1 antibody	(67)
VV-SCFV-TIGIT	Breast, colon and hepatic cancer	SCFV of *anti-TIGIT* and *anti*-*PD-1*	(68)
Western Reserve (WR) OVV	Melanoma Fibrosarcoma	scFv, Fab and antibody forms of a hamster monoclonal IgG (namely J43) recognizing the murine PD-1	(69)
GLV-1h68	Soft-tissue Sarcoma	Recombinant rat anti-PD-1 protein	(70)
JX-594	Murine bladder, Colon cancer, Lung	Anti-PD-1 antibody	(71)
CF33-hNIS-antiPDL1	PDAC	*Anti-PD-L1*	(72)
OVV-MnSOD	Lymphoma	Anti-PD-L1 antibody	(73)
vvDD	Colon and ovarian	Anti-PD-L1 antibody	(74)
JX	Colon	Anti-PD-1 antibody	(75)

A similar study suggested the use of mJX-594 (JX), a vaccinia virus equipped with GM-CSF (cytokine), as an approach to remodel the tumor microenvironment and augment sensitivity to αPD-1 and/or αCTLA-4 immunotherapy. The administration of JX resulted in changes in the tumor microenvironment as indicated by the infiltration of T-cells into the tumor and the high expression of genes associated with immunity. It not only increases the infiltration of CD8+ T-cells within the tumor but also enhances abscopal effects in distant tumors. Combining intratumoral JX with systemic αPD-1 or αCTLA-4 enhanced the anticancer immune response. Notably, the strongest anticancer immunity was exhibited when including JX, αPD-1, and αCTLA4, causing complete tumor regression and prolonged overall survival (77).

### ICI immuno-oncolytic virotherapy

Oncolytic viruses can be genetically engineered to create immuno-oncolytic viruses (IOVs), themselves encoding immunomodulatory factors (78). IOVs can be used for the localized expression of immune-modulating target proteins, obviating the need for separate immunotherapy following virotherapy. One type of immuno-oncolytic virotherapy involves the insertion of the anti-PD-L1 antibody (anti-PD-L1) expression cassette into an OV. This OV then both directly mediates cancer killing and induces the production of the anti-PD-L1 antibody. This anti-PD-L1 decreases immune inhibition, enhancing the treatment’s oncolytic efficacy by improving anti-tumor immune cell function (79).

**Figure 2:**
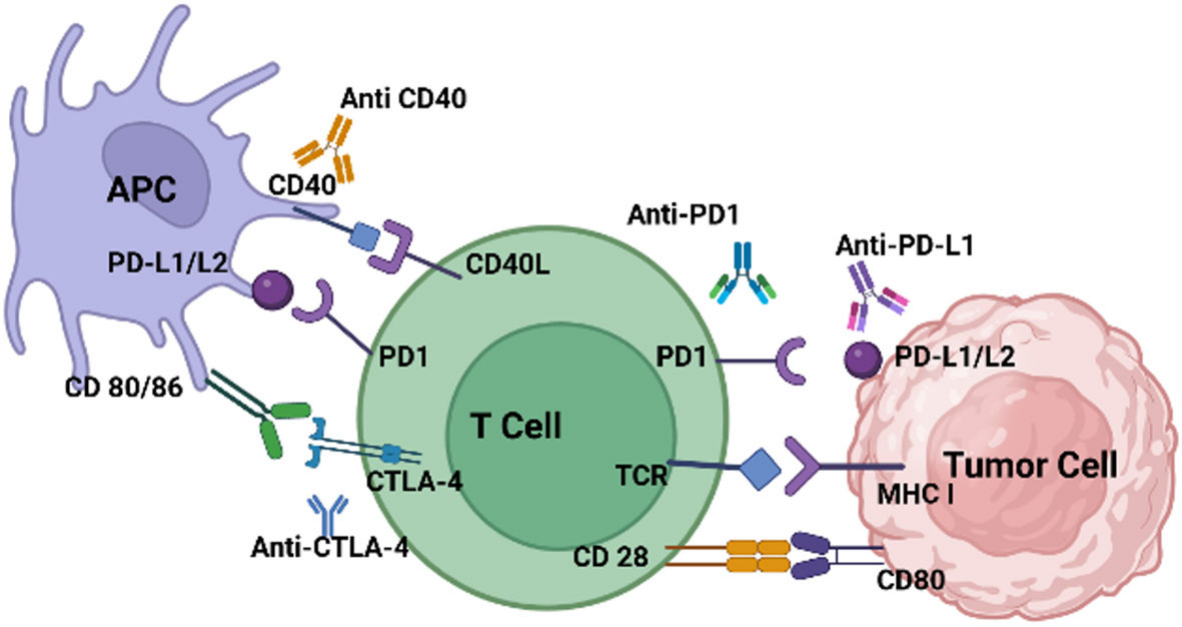
T-cells are inhibited when immune-checkpoints bind to ligands on tumor cells and APCs. PD-L1/L2, CD40, CD80/86, are expressed on tumor cells or APCs. Their expression is induced and maintained by many factors. PD-1, CTLA-4, CD40L and CD28 are expressed on exhausted effector T-cells. CTLA-4, which interacts with its ligands B7-1/CD80 and B7-2/CD86, or PD-1, binding to its ligand PD-L1, generate inhibitory signals, dampening T-cell immune responses. These receptors, CTLA-4 and PD-1, serve as targets for ICIs like anti-CTLA-4, PD-1, and PD-L1 aiming to decrease immune cell inhibition.

**Figure 2** illustrates the variety of ICI ligands on tumor cells and antigen-presenting cells (APCs), along with their cognate receptors on T-cells. The higher the ICI expression on the tumor cell’s surface, the greater the chance of response to immunotherapy treatments. ICIs that target the PD-1/PD-L1 axis have been effective in solid tumors, such as non-small cell lung cancer (NSCLC), with high levels of PD-L1 expression and active lymphocytic infiltrates (80). Unfortunately, ICIs that depend on single targets have failed in pancreatic ductal adenocarcinoma (PDAC) patients, even those with tumors overexpressing PD-L1. Low antigen recognition limited cytotoxic T-cell recruitment, and aberrant signaling of co-stimulatory immune checkpoints have been identified as critical barriers to the success of immunotherapies in PDAC (81). CF33-hNIS-antiPDL1 is an OV genetically engineered using the potent chimeric orthopox virus backbone CF33 to contain the human Sodium Iodide Symporter (hNIS) and anti-PD-L1. Unmodified CF33 has previously been demonstrated to be safe and well-tolerated at doses several magnitudes lower than other OVs currently in preclinical and clinical studies in pancreatic cancers by the authors (82). CF33-hNIS-anti-PD-L1 was shown to lyse PDAC cells in a dose-dependent manner, achieving >90% cell killing by day three. Infected cells were shown to produce bioactive anti-PD-L1, which blocked the PD-1/PD-L1 interaction. *In vivo*, a single dose of the engineered virus both reduced tumor burden and prolonged the survival of treated mice (72).

**Figure 3:**
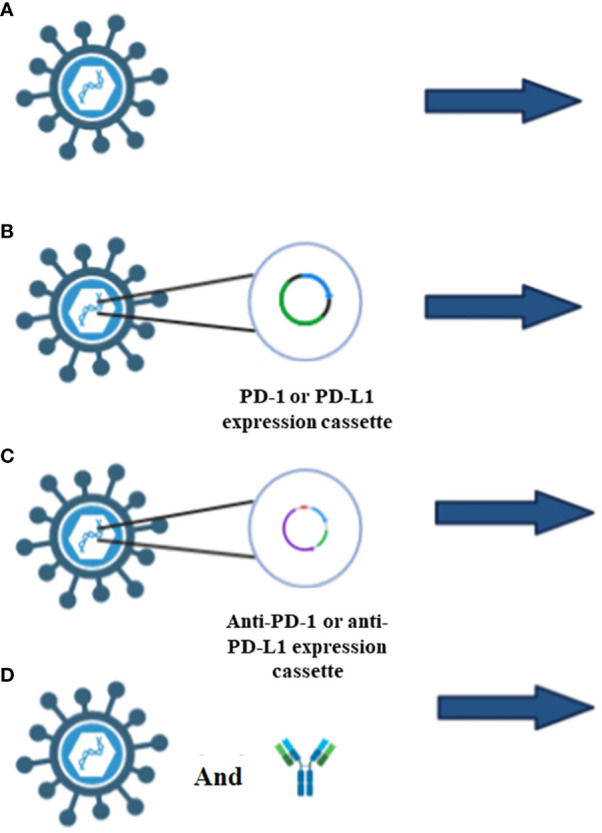
Different types of ICIs applications. **(A)** OVV injection alone increases the ICIs expression which is due to the increase of interferon γ levels through the JAK/STAT pathway. **(B)** Insertion of PD-1 or PD-L1 in OVV induce the overexpression of ICIs which increase the success rate of immunotherapy. **(C)** Insertion of anti-PD-1 or anti-PD-L1 antibody, block the interaction of ICIs with T-cells, therefore t-cell can inhibit the tumor cells. **(D)** Injection of OVV and anti-PD-1 antibody or anti-PD-L1 antibody in a designed regimen not only recruit immune cells, but also inhibit the interaction of ICIs with T-cells.

Researchers inserted two genes encoding the anti-PD-1 antibody (anti-PD-1) and the anti-human-tumor necrosis factor receptor superfamily member 9 (TNFRSF9 also known as CD134 or 4-1BB), into an OV named ΔTK-ARMED-VACV. ΔTK-ARMED-VACV was shown to have tumor-specific cytotoxic properties and the antibody genes were encoded without affecting viral replication. In an *in vivo* study, ΔTK-ARMED-VACV inhibited tumor growth and increased IFN-γ in treated mice (66). T-cell immunoglobulin and ITIM domain (TIGIT) is an immune checkpoint that inhibits the immune system’s activity. In 2021, Zue et al. designed VV-scFv-TGIT, an OVV encoding a single-chain variable fragment (scFv) targeting TIGIT. VV-scFv-TIGIT’s anti-tumor efficacy was explored alone and in concert with PD-1 or lymphocyte-activation gene 3 (LAG-3) blockades. Injection of VV-scFv-TIGIT into mouse models showed that this virus specifically replicates in tumor cells and converts cold to hot tumors by recruiting CD8+ T-cells to the site. The VV-scFv-TIGIT group expressed IFN-γ, TNF-α, Grant B, IL-6, and IL-10 more than the wild-type VV or PBS control groups. VV-scFv-TIGIT increase PD-L1 expression on the surface of tumor cells which can increase tumor immunogenicity and response to immunotherapy. PD-1 or PD-L1 can be inserted into OVV to induce ICI overexpression, and anti-PD-1, anti-PD-L1, or their associated antibodies can block the interaction of ICI ligand and receptor to allow T-cells to remain active to remove cancer cells more effectively (**Figure 3**). In mice, encoding 3 forms of mPD-1 binders including whole antibody (mAb), fragment antigen-binding (83), or single-chain variable fragment (scFv) into Western Reserve (WR) oncolytic vaccinia virus improved the oncolysis ability of this virus. Importantly, intra-tumoral injection of this virus increased mAB secretion 1900 times compared to subcutaneous injection (69).

### Cytokine-expressing OVs

Cytokines play a pivotal role in orchestrating the immune response against tumors, with various interleukins contributing to the elimination of cancerous cells. Interleukin-2 (IL-2) is a potent cytokine that stimulates the proliferation and activation of cytotoxic T-cells and natural killer cells, enhancing their ability to recognize and eliminate tumor cells (11). rVVDD-hIL2 is a genetically modified oncolytic vaccinia virus armed with human interleukin-2 (hIL2) (9). This recombinant virus effectively infects and kills tumor cells while expressing increasing levels of hIL2, indicating its potential as a promising vector for cancer treatment. The insertion of hIL2 does not compromise viral replication capacity, highlighting the therapeutic potential of this approach (84).

Interleukin-12 (IL-12) promotes the differentiation of T-cells into cytotoxic T lymphocytes (CTLs) and enhances their anti-tumor activity. Oncolytic vaccinia virus delivering tethered IL-12 converts “cold” tumors, characterized by a scarcity of T-cell tumor infiltration, into “hot” tumors, with an abundance of tumor-infiltrating T-cells, enhancing immune responses without systemic toxicity and cytokine release syndrome (85). An oncolytic vaccinia virus incorporating hIL-7 and mIL-12 genes exhibited antitumor activity by increasing the inflammatory immune status and rendered the tumors susceptible to immune checkpoint blockade. The activation of immune responses was observed both in treated tumors and untreated distant tumors found on the animal’s other flank. These encouraging outcomes, validated in humanized mice with human cancer cells, underscore the potential for further investigation in individuals with non-inflamed solid tumors (22).

IL-15 supports the development and function of NK cells and memory T-cells, contributing to sustained anti-tumor immunity. In one study, OVVs were developed from the VV Lister strain from the Institute of Virus Preparation, Moscow, Russia (LIVP) that express interleukin-15 (IL-15) or its receptor subunit alpha (IL-15Rα). Using murine colon and breast carcinoma models, the oncolytic activity of these new variants was evaluated, for each virus alone and both in combination. Shakiba et al. demonstrate that the combination of these recombinant variants promoted the generation of the IL-15/IL-15Rα complex. *In vivo*, mice bearing syngeneic 4T1 tumors had significant tumor regression and increased survival following treatment with the combination of LIVP-IL15-RFP and LIVP-IL15Ra-RFP, lymphocytes were recruited to tumor sites, and no harmful effects were observed in the liver or spleen (40).

The IL-1 cytokine family, including IL-36α, IL-36β, and IL-36γ, although less studied in the context of tumor immunity, has been implicated in inflammation and may have potential consequences for modulating the immune response against cancer. IL-36γ has previously been shown to promote interferon-γ (IFN-γ) production, increase type 1 immune responses in the TME, and promote antitumor immune responses (86). Several VV genes have been implicated in mediating the function of IL-1 family members, making these cytokines attractive targets when modulating OVV immunogenicity (54). These genes include A46R, a putative IL-1 antagonist, B13R, also known as SPI-2, inhibiting the enzyme converting pro-IL-1β to IL-1β, and B15R, a soluble IL-1β receptor (35, 63). Yang et al. developed an OVV expressing IL-36γ (IL-36γ -OV).

An OVV expressing IL-36γ (IL-36γ-OV) developed by Yang et al. showed significant therapeutic efficacy in multiple murine tumor models. Additionally, IL-36γ-OV modulated the TME, promoting dendritic cell and lymphocyte infiltration and reducing myeloid-derived suppressor cells and M2-like tumor-associated macrophages, into the tumor (87).

Thus, these engineered viruses encoding various cytokines not only induce direct oncolysis but are able modulate the tumor microenvironment, and may lead to increased infiltration of immune cells, reduced immunosuppressive elements, and increased differentiation of T-cells into effector cells. The combination of OVs with diverse cytokines showcases a multifaceted approach, harnessing both viral oncolysis and cytokine-mediated immunomodulation, holding great potential for advancing cancer treatment strategies.

### IFN therapy

The introduction of IFN-γ genes into OVVs is strategically designed to enhance the OVVs’ oncolytic potential. This genetic modification aims to amplify the anti-tumor effects of OVVs, capitalizing on the immunomodulatory properties of interferon-beta (IFN-γ). Interferons play a crucial role in the body’s innate immune response, and IFN-γ, in particular, has been recognized for its ability to regulate immune functions and exhibit antiviral and anti-tumor activities (88). IFN-γ genes inserted into OVVs can increase the anti-tumor effects but also increase virus inactivation in non-cancerous cells. Preclinical testing of this combination therapy used the VV B18R deletion mutant as a backbone for the expression of IFN-γ. This newly engineered OVV showed IFN-dependent cancer selectivity and efficacy in both tumor cells and tumor-associated vascular endothelial cells *in vitro*, and tumor targeting and efficacy in *in vivo* mouse models (89).

### Chemotherapy

Combining vaccinia virotherapy with chemotherapy agents is a promising approach to cancer treatment. For example, a combination of VV with paclitaxel produced a synergistic effect mediated by type I interferon, secreted soon after infection, and high-mobility group protein B1, secreted after cell death (90). The sorafenib and VV combination has shown promising anti-tumor results in some models and patient trials (31, 91, 92). In a mouse model of lung adenocarcinoma, CPA and GLV-1h68 had synergistic anti-tumor effects. In a study of human colorectal adenocarcinoma, OVV synergized with irinotecan to decrease tumor cell viability. Combination therapy significantly improved survival over either monotherapy. Cyclophosphamide (CPA), and GLV-1h68 have synergistic anti-tumor effects on PC14PE6-RFP xenografts. Protein profiling of tumor lysates for the untreated, CPA-, GLV-1h68- and combination treatments showed that host-derived pro-inflammatory cytokines and chemokines, such as eotaxin, MIP-1β, MCP-1, MCP-3, MCP-5, TNF-α, and MPO, are upregulated in tumor tissues after viral infection and combination therapy OVVs may synergizes with irinotecan (CPT-11) in human colorectal adenocarcinoma. This combination therapy significantly decreased tumor cell viability compared to each method used as a monotherapy. It is hypnotized that the sequence of administration of chemotherapy and virotherapy influences the interactions between these two treatment methods (93).

Many cancer cells secrete high levels of apoptosis-inhibiting proteins (IAPs) and escape from apoptosis in this way. Mitochondrial protein Smac inhibits IAPs, such as XIAP, and the activity of caspases, thereby inducing apoptosis in cancer cells. In the oncolytic virus VV-Smac, the Smac gene is inserted. When used in combination with chemotherapy, in addition to causing the Smac protein’s expression, VV-Smac reduced cancer cells’ survival (94).

The therapeutic combination of mpJX-594, a replication-competent VV, and sunitinib increases vascular pruning, leakage and recruitment of CD8+ T-cells; and decreases the invasion and metastasis of cancer cells. This synergy functions with sunitinib reducing tumor vasculature, subsequently cutting tumor angiogenesis, which triples the effect of the mpJX-594 compared to virus therapy alone (95).

Doxorubicin-resistant ovarian cancer cells (A2780-R), are resistant to oncolysis by OVV. The mechanism of this resistance is inhibition of OVV replication by STAT3 protein kinase inhibitors in resistant cells. To overcome this 2-sided resistance, combination therapy of OVV and trametinib was used, which significantly reduced xenograft tumor growth (96). In a case report study, pretreatment of a person resistant to chemotherapy with the OVV GL-ONC1 laparoscopically and a combination of chemo drugs (paclitaxel, carboplatin, bevacizumab) increased the response to treatment (29). In patient who have had surgery as the only treatment, without prior chemotherapy, the injection of a single dose of OVV can increase the response to treatment by secreting IFNα and other chemokines (97). **Table 3** shows different combinations of OVV strains with chemotherapy drugs.

**Table 3:** Chemotherapy drugs in combination with OVV therapy.

Chemotherapy drug	Vaccinia virus strain	Study	Sample	Result	Ref.
**Paclitaxel**	vvDD-luciferase	*In-vitro* *In-vivo*	Human colorectal cancer cell lines: HCT-116 Human colorectal cancer cell lines: MCF-7 human ovarian cancer cell line: UCI-101	Synergistic effect due to IFN mediation	(90)
**Sorafenib**	JX-594	*In-vitro* *In-vivo* Clinical	HepG2, PLC/PRF/5, A2780, (ovarian) HCC lines SNU423, SNU475 and SNU449 HCC patients	JX-594 sensitize tumors to subsequent therapy with VEGF/VEGFR inhibitors	(92)
**Cyclophosphamide**	GLV-1h68	*In-vitro* *In-vivo*	PC14PE6-RFP	Up regulation of cytocine and chemokine	(98)
**Irinotecan**	vvDD	*In-vitro* *In-vivo*	Human colorectal adenocarcinoma: HT29 and murine colorectal adenocarcinoma: DLD1 and MC38 murine sarcoma cell lines: 24-JK	Combination therapy significantly improved survival over either monotherapy	(99)
**Nabpaclitaxel+ Gemcitabine**	GLV-1h68	*In-vitro*	Human pancreatic adenocarcinoma cell lines: AsPc-1, BxPc-3, MIA-PaCa-2, and Panc-1 resulted in enhanced tumor cell killing in ABxPc-3 and MIA PaCa-2	Enhanced tumor cell killing in ABxPc-3 and MIA PaCa-2 compared to monotherapy	(93)
**Gemcitabine**	OVV-Smac generated from the Western Reserve strain (WR)	*In-vitro*	The human pancreatic cancer cell lines SW1990, BXPC-3 and PANC-1	Enhanced tumor cell killing due to stimulation of apoptosis	(94)
**Sunitinib**	Mouse-prototype JX-594 generated from the Western Reserve strain (WR)	*In-vivo*	Tumor-bearing RIP-Tag2 transgenic mice bearing pancreatic neuroendocrine tumors	Amplification of virus effect by the multitargeted kinase inhibitor sunitinib	(95)
**Combination of paclitaxel carboplatin and bevacizumab**	GL-ONC1	Case report	A heavily pretreated ovarian cancer patient	It remained unknown if clinical response was due to the combination of OVV and chemotherapy or due to subsequent chemotherapy regimen	(29)
**Cytarabine**	oVV-ING4 which expresses the inhibitor of growth family member 4	*In-vitro* *In-vivo*	(AML) and (CML) cell lines	Induction of apoptosis *in-vitro* and *in-vivo*	(100)

Direct delivery of chemotherapy drugs to a specific site can be achieved using the isolated limb perfusion (ILP) technique. This method involves the insertion of a cannula into the blood vessels leading to the tumor area, isolation of the organ from systemic circulation through the use of a tourniquet, and high-dose chemotherapy administration. Standard ILP treatment employs melphalan with tumor necrosis factor alpha (TNF-α) and has been used to treat advanced extremity sarcoma and in-transit melanoma (101). The ILP of GLV-1h68 results significant viral deposition in the tumor with evidence of viral replication within the tumor. At the same time, the virus cannot damage healthy cells. The combination of GLV-1h68 with melphalan ILP intensified the effect of virus treatment (102).

### Radio therapy

Radiotherapy has a well-established role in local disease control and may be given either pre- or post-operatively. Combining radiotherapy and OVs has demonstrated potential in cancer treatment. The synergistic effects of radiotherapy and OVV therapy can be seen in many studies. Expression data obtained from a combinatorial regimen of radiotherapy and OVV therapy demonstrated that this synergy is not due to increased viral replication. Rather, it is mediated through the induction of intrinsic apoptosis. One study demonstrated that GLV-1h68 therapy downregulated the anti-apoptotic BCL-2 proteins MCL-1 and BCL-XL as well as downstream inhibitors of apoptosis, resulting in cleavage of effector caspases 3 and 7. In the ILP rat model, the combination of OV and radiotherapy significantly delayed tumor growth and prolonged survival compared to single-agent therapy (103). The efficacy of OVV and radiotherapy in an *in vitro* assessment of head and neck cancer was shown to be dose- and time-dependent. In CD-1 nude mice, this same combination induced caspase activity and increased long-term regression (104). A combination of the OVV GLV-1h151 and radiotherapy in AsPC-1, a human pancreatic adenocarcinoma cell line, xenografts in mice inhibited tumor growth and had no toxicity to the mice (105). Combining radiotherapy and OVV increased necrosis and apoptosis in tumors and the ensuing release of DAMPs. This combination enhanced the *in vivo* anti-tumor effect and increased splenic CD4+Ki-67+ helper and CD8+Ki-67+ cytotoxic T lymphocytes, and tumor-infiltrating CD3+CD4+ helper and CD3+CD8+ cytotoxic T lymphocytes. Conversely, tumor-infiltrating regulatory T-cells were decreased using this same combination of OVV and high-dose hypofractionated stereotactic body radiotherapy (106). In a first-phase trial of intravenous OVV GL-ONC1 with cisplatin and radiotherapy, the 19 patients with advanced head and neck cancer included had no disease progression for 30 months following treatment, with an overall survival rate of 74.4% reported. However, due to the intravenous injection of therapeutics, virus delivery to the tumor was challenging, and some patients reported grade 1, 2, and 3 side effects (32). Since VV has a DNA genome, there is concern that it may be destroyed or modified following radiotherapy. To study this effect, Wilkinson et al. injected doses of GLV-1h68 by ILP to rats and then treated them with external beam radiation therapy (EBRT). This study shows that not only is the DNA of GLV-1h68 resistant to EBRT, but this combination has additive and possibly synergistic effect (103). The reason for this result in this study could be that the harmful effects of radiation on the virus were controlled in terms of time. It seems that prescribing a suitable program of virus injection and radiotherapy or any other combined treatment can not only limit the effects of the two methods on each other, but also can have more oncolysis effects.

### CAR-T cells therapy

Chimeric antigen receptor (CAR) T-cell therapy has recently transformed the treatment of refractory hematological cancers, including acute lymphoblastic leukemia and chronic lymphocytic leukemia. CAR-T cell therapy entails the *in vitro* design, modification, and amplification of T-cells such that they recognize tumor cell surface antigens using the T-cell surface transduced CAR structure. This allows these CAR-T cells to move into the tumor microenvironment of “hot” tumors and to kill cells displaying the cognate antigen. In 2017, the FDA approved CD-19-specific CAR-T cells to treat refractory B-cell lymphomas (107). Despite this advance, in patients with multiple solid tumors, only minor and transient responses were observed, perhaps due to poor tumor penetration and impaired function of these T-cells in the “cold” tumor environment. OVs are capable of potential synergy with CAR-T cell therapies as they can promote the migration, proliferation, and activation of T-cells, particularly when they are engineered to deliver immunostimulatory cytokines, T-cell attracting chemokines, or immune checkpoint-targeting molecules (108).

T-cell trafficking depends on the correct combination of cell-secreted chemokines and chemokine receptors on the effector cell. Often, tumors produce only nominal amounts of chemokines, which result in the immunologically “cold” tumor phenotype with very few effectors successfully reaching the tumor (109). Modified OVV (VV.CXCL11) which is produced by inserting the CXCR3 ligand on CXCL11 increase T-cell trafficking into tumors. VV.CXCL11 has the ability to recruit total and antigen-specific T-cells into the TME. As well, VV.CXCL11 significantly enhanced the anti-tumor efficacy of recruited T-cells as compared to the direct delivery of CXCL11 by CAR-T cells (108, 109). Similarly, engineered OVV (OV19t) that express a non-signaling, truncated CD19 (CD19t) protein, enable CAR-T cell to target the infected cells. *In vitro*, OV19t infection of tumor cells result in *de novo* cell surface CD19 expression prior to the virus-mediated lysis of tumor cells, with co-cultured CD19-CAR-T cells secreting cytokines and exhibiting potent cytolytic activity against infected tumor cells. *In vivo*, OV19t aided tumor control following the administration of CD19-Car-T-cells and local immunity against tumor cells with tumor infiltration of both endogenous and injected CAR-T cells. The CAR-T cell-mediated tumor killing also caused OV19t to be released from dying cells which propagated tumor expression of CD19t, allowing greater viral spread and a more widespread response (110).

### OVV armed with bi-specific T-cell engagers

Bi-specific T-cell engagers (BiTEs) are a type of immunotherapy designed to enhance the body’s immune response against cancer cells. These molecules are engineered to simultaneously bind to T-cells and cancer cells, bringing them into close proximity. This dual binding allows for the formation of the immunological synapse where activated T-cells can secrete perforin and other granzymes leasing to cancer cell lysis (111). OVVs can be engineered to encode BiTEs. T-cell engager CD3-scFv provides an alternative approach for the engagement of T-cells for cancer immunotherapies. In 2014, Yu et al. constructed one such VV encoding a BiTE consisting of a scFv for CD3 and one specific for EphA2, a tumor cell surface antigen, named EphA2-TEA-VV. *In vitro* analyses revealed similar replication and cancer cell lysis when compared to the GFP-expressing double-deleted WR control. Similarly, EphA2-TEA-VV directed T-cells towards cancer cells, activated T-cells through secretion of IFN-γ, IL-2, and binding to CD3, and induced bystander killing of noninfected tumor cells. *In vivo* analysis of an A549 NSCLC xenograft model in SCID-Bg mice demonstrated EphA2-TEA-VVs potent antitumor effect compared to the GFP control. Treatment with both EphA2-TEA-VV and unstimulated peripheral blood mononuclear cells from healthy donors synergized to have stronger antitumor effects compared to each treatment used as a monotherapy. Consequently, this suggests that arming oncolytic VVs with T-cell engagers has the potential to improve therapy potency by boosting T-cell activation and promoting broader destruction of tumor cells, including those not infected, offering a promising strategy for advancing oncolytic virus therapy in cancer treatment (112).

Yu et al.’s team also created an oncolytic vaccinia virus armed with a BiTE targeting murine CD3 and fibroblast activation protein (FAP), known as mFAP-TEA-VV. Their primary goal was to address safety concerns linked to FAP-targeted immunotherapies related to off-target activation, and which have shown significant antitumor effects like (112). Similarly, to EphA2-TEA-VV, mFAP-TEA-VV demonstrated similar replication ability and oncolysis as an unmodified VV control, and was also capable of triggering bystander killing of uninfected FAP-expressing cells in co-culture assays with murine T-cells. In an immunocompetent B16-F10 model *in vivo*, mFAP-TEA-VV increased viral load within the tumor and has strong antitumor effects compared to the VV control. Notably, the enhanced spread of the mFAP-TEA-VV virus was associated with the degradation of the tumor stroma, indicating a correlation between improved viral distribution and the destruction of the supportive tissue around the tumor (113).

The “double-deleted” Western Reserve vaccinia virus (vvDD), was engineered to express an anti-FAB/CD3 BiTE. This demonstrated improved viral replication and promoted the infiltration of active T-cells in the melanoma syngeneic models, tumor regression was proven. Furthermore, anti-FAP/CD3 BiTE-expressing Ad ICOVIR15K showed better antitumor responses and intratumoral T-cells. In addition, aside from the BiTE approach, VV that encoded anti-FAP expressed moderate regressions in tumor growth compared with unarmed VV. However, a notable reduction of cancer associated fibroblasts (CAF) was observed. This moderate regression in antitumoral activity can be caused using a cytotoxic FAP-blocking antibody over the cytotoxic anti-FAP BiTE. The FAP-blocking antibodies did allow the CAF to regrow and restore the TME (114).

In 2022, Wei et al. developed VV-EpCAM BiTE to enhance antitumor immunity in solid tumors by modulating the immunosuppressive TME. This recombinant virus secretes a BiTE against epithelial cell adhesion molecule (EpCAM), a solid tumor-associated antigen, and CD3. VV-EpCAM BiTE effectively infected and lysed cancer cells. The binding of EpCAM-expressing malignant cells and CD3ϵ on T-cells, mediated by the secrete EpCAM BiTE, activated T-cells and led to the release of IFN-γ and IL-2. When administered intratumorally, VV-EpCAM BiTE significantly improved antitumor activity, particularly in tumors with high EpCAM expression. This treatment also increased TME immune cell infiltration, reduced CD8+ T-cell exhaustion, and enhanced T-cell-mediated immune activation (115).

Lei et al. engineered an oncolytic vaccinia virus expressing CD19-specific BiTE (OVV-CD19BiTE). When compared to the non-engineered type, OVV-CD19BiTE can recruit more CD3, CD8, and naïve CD8 T subpopulations to tumor tissues, while retaining similar ability to replicate in and lyse tumor cells (116).

### Photodynamic therapy

Photodynamic therapy (PDT), is a clinically approved therapy for solid tumors based on the photo-activation of a tumor localizing agent, the photosensitizer (60), resulting in the generation of cytotoxic singlet oxygen (^1^O_2_) [37 (60). In this treatment modality, a PS is combined with an immune protein to allow PS delivery to cancer cells. When light is applied, the PS kills cancer cells and causes the generation of an immune response which can lead to further cancerous cell death. The second-generation, chlorin-based 2-(1-hexyloxyethyl)-2-devinyl pyropheophorbide-a (HPPH) sensitizer exhibits favorable photophysical and pharmacokinetic properties in clinical trials, as compared to the porphyrin-based photosynthesizer Photofrin (117). In 2004 pre-clinical studies, HPPH-PDT was shown to result in varying patterns of vascular, cellular and inflammatory responses with long-term tumor control rates depending on the specific treatment regimen employed (118). HPPH-sensitized PDT may have therapeutic activity in combination with a TK- and VGF-deleted VV. This VV expressed enhanced green fluorescent protein, EGFP, used as a marker to track NXS2 neuroblastomas established in syngeneic mics and human FaDu xenografts in athymic nude mice. The combination of PDT and OVV was the most effective at controlling primary and metastatic tumor growth compared to either treatment used as a monotherapy. One dosage combination even caused a decrease in tumor volume in both NXS2 and FaDu tumors within six weeks of treatment. Since PDT leads to the disruption of tumor vascularization, the virus titer in the tumor was higher in the combination than in oncolytic monotherapy, which indicates that these two treatment methods combined synergistically and did not negatively impact each other (118).

### RNA therapy

RNA therapy is a type of therapeutic method based on RNA molecules, in which RNAs are used to manipulate the expression and activity of target molecules. The first RNA-based drug was used in the 1990s, during which mRNAs were injected into mice to target protein production. Recently, micro RNAs, single-stranded mature miRNAs of ~22 nucleotides, have been used to treat diseases, especially cancer. In this method, miRNAs act as oncomiR by targeting tumor suppressor genes, or as tumor suppressor by targeting oncogenes. miRNAsbind to complementary sequences in the 3’-untranslated region (UTR) of a messenger RNA (mRNA), so they can repress gene expression post-transcriptionally. Therefore, by combing miRNA therapy and OVV therapy, the virus’ pathogenic genes can be removed to increase the safety profile of the OV therapy. For example, B5R protein downregulation mediated by let-7a miRNA was shown to decrease viral pathogenicity and impair the oncolytic activity of VV. This let-7a miRNA-regulated VV (MRVV) is capable of selectively replicating and inducing oncolysis of tumor cells while displaying no toxicity to healthy cells. B5R expression and MRVV replication depended on infected cells’ endogenous let-7a expression level. The oncolytic potentials of the B5R-negative LC16m8Δ and B5R-positive LC16mO viruses were compared in mouse models with human cancer xenografts using intratumoral administration of the MRVV. While both viruses decreased tumor size 18 days post-infection, LC16mO-treated mice died or were sacrificed on days 21-28 following severe viral toxicity symptoms, including weight loss and pock lesions on tails, paws, faces, and other areas of the body, while LC16m8Δ mice did not exhibit these same symptoms. This group showed that this transgene insertion did not affect let-7a-regulated oncolytic activity, MRVV replication was inhibited in normal tissues, and MRVV reduces viral pathogenicity while maintaining oncolytic activity (119).

OVV therapy was shown to be more effective in paclitaxel-resistant KFTX ovarian cancer cells than KFlow paclitazel-sensitive cells. The long non-coding RNA (lncRNA) urothelial carcinoma-associated 1 (UCA1) is overexpressed in paclitaxel-resistant cells, and UCA1 expression was shown to correlate to the oncolytic effects of OVV in various primary ovarian cancer cell lines. This suggests that UCA1 is involved in regulating OVV’s oncolytic effects. UCA1 also enhanced the cell-to-cell spread of OVV via the activation of Cdc42, a Rho GTPase, resulting in better therapeutic outcomes in ovarian cancer (120). Similarly, it has been shown that UCA1 significantly increases OVV cell-to-cell spread in colorectal cancer. UCA1 inhibited miR-18a and miR-182, promoting the Cdc42 activation that enhanced OVV spread via filopodia formation (120).

### Hormone therapy

Aldosterone is a type of mineralocorticoid hormone that controls the balance of water and salts in the kidneys by retaining sodium and releasing potassium from the body. In the phase I hepatoma clinical trial of JX-594, an OVV derived from the Wyeth strain, Park et al. noted viral replication in most patients, with remarkably more detected in cancer patients with severe ascites and peripheral edema. Ascites is the abnormal accumulation of fluid within the peritoneal cavity that occurs due to advanced cancer. This abdominal fluid sequestration leads to further fluid retention by the kidneys due to the stimulatory effects of hormones such as aldosterone, which can cause further peripheral edema. It is subsequently assumed that aldosterone levels increase in these patients and may increase the proliferation of tumor cell lines (31). In another study, viral replication significantly increased with simultaneous JX-594 and aldosterone treatment in A2780, PC-3, and HepG2 cells but not U2OS cells. The differential timing of aldosterone treatment altered viral replication differently in each cell line. The aldosterone-receptor inhibitor spironolactone inhibited JX-594 entry across the cell’s plasma membrane. Similarly, viral entry was significantly decreased by treatment with 5-N-ethyl-N-isopropylamiloride (EIPA), an Na^+^/HA^+^-exchange inhibitor, but restored following aldosterone treatment (121). These findings suggest interactions between OVV therapy and hormone levels such that synergy may be observed between oncolytic virotherapy and hormone therapies.

## Shielding OVVs from the innate and adaptive humoral immunity

One limitation of the therapeutic use of OVs is viral inhibition by the immune system. A range of different OV delivery methods have been explored, including both intratumoral injection and systemic delivery. Intratumorally injected OVs have demonstrated success but allow only the treatment of easily accessible solid tumors, relying on viral replication within the TME and subsequent dissemination to distant sites to treat metastases. This replication and dissemination is often ineffective due to immune responses against the OV. Systemic OV delivery allows the treatment of both primary tumors and any metastases, whether overt or undiagnosed, and is an attractive option for treating metastatic and inaccessible solid tumors and blood cancers. Vaccine-based OVs induce a strong immune response within the tumor to aid in killing tumor targets, but this same immune response may sometimes interfere with the virus’ oncolytic activity. A primary focus of OV research is thus how to protect the therapeutic virus from the patient’s immune system (122). During an infection, viral entry into cells increases intracellular interferon secretion, leading to inflammation. The secretion of innate immune cells, such as NK cells and phagocytes, increases during the inflammatory response. Concurrently, viral antigens enter lymph nodes and, with the help of antigen-presenting cells such as DCs, activate the adaptive immune response of B and T-cells. Activated B-cells differentiate into plasma cells that secrete antibodies that disrupt the viral life cycle by preventing entry into susceptible cells. T-cells directly attack and destroy the virus through cytotoxic effects. Similarly, systemic VV delivery is limited by pre-existing immunity. For OVV, as worldwide immunization with VV was undertaken during the eradication of smallpox, many individuals who are currently developing cancer present with immunity to this virus.

Complement is a crucial component of the innate immune system’s first line of defense, targeting foreign pathogens for opsonization, neutralization, phagocytosis, and clearance from the circulatory system. In some cancer patients, neutralizing antibodies due to smallpox vaccination can limit the efficacy of OV therapy. Natural barriers in the blood, including these antibodies and complement, will likely limit the efficacy of repeated intravenous OV administrations. Depleting complement proteins using cobra venom factor (CVF) improved OV delivery and infection of tumors in rats (123) Additionally, a combinatory strategy of complement inhibition and OV therapy in cynomolgus macaques demonstrated enhanced tumor control at early time points (124).

Given the promising results of existing oncolytic therapies and research, it is clear that a balance must be struck between antiviral and anti-tumor immunity. This balance may be created by injecting sufficiently high initial amounts of the virus, combining OV therapy with other immunotherapies, and methods that shield the virus from the immune system, since increasing OV doses is not a good option as it may cause symptoms associated with viral disease in patients, especially those already immunocompromised at the start of treatment (125). Local intratumoral virus injection can largely overcome this problem and deliver the virus to the tumor cells before being targeted by the immune system. Below, various methods are discussed for keeping the OVV from the immune system.

### Coatings

One way to protect the therapeutic virus from the immune system is to use physical shields. These physical protectors can be cell-derived nanovesicles, liposomes, or chemical polymers. Polylactic-co-glycolic acid (PLGA) is a biocompatible and biodegradable synthetic material widely used in studies of nanotechnology and drug delivery. PLGA nanofiber has been used as a stent for the targeted delivery of an OVV to a CRC tumor and was studied using *in vitro* cell cultures and in an *in vivo* animal tumor xenograft., In CT-26 cells, the infectivity and anti-tumor activity of OVVs released from the PLGA membrane were preserved, with cell viability decreasing in a dose-dependent manner. Similarly, this study showed that the embedded virus was continuously released over 48 hours (126). The use of virus coatings, however, may have limitations that impede use. For example, coatings may prevent OVs from accessing and affecting tumor cells and present barriers to commercialization due to greatly increased costs (127).

### Cellular carriers

Another way to protect viruses from the immune system is via the use of cell carriers. In this method, carriers are loaded with the virus to allow their undetected transport throughout the body. One example where the use of cell carriers may be suitable for the delivery of OVs is in brain cancers. Due to the presence of the blood-brain barrier, OVV has difficulty accessing brain tumors. However, the influx of blood and cerebrospinal fluid into the brain following tumor resection can be hijacked, such that, using cell carriers, oncolytic virotherapeutics are delivered to the brain. These cell carriers can be derived from a variety of cells. For example, they may come from stem cells such as mesenchymal stromal cells (MSCs). Extracted MSCs from adipose tissue, called adipose tissue-derived stem cells (ADSCs), were infected with the VV LIVP strain and co-cultured with blood mononuclear cells. The viability of these cells was then checked *in vitro*. The results show that co-culture of OVV with these mesenchymal cell carriers (the ADSCs) increased OVV efficacy (128). These cell carriers face limitations to their use in clinical studies due to allogeneic responses from NK cells and adaptive T-cells, and since therapeutic efficiency may be reduced due to the removal of the ADSCs by the patient’s immune cells. Cell carriers may also be derived from solid tumors. Due to potential risks, injecting these types of carriers into patients has yet to progress into clinical trials. In addition to taking advantage of both OVV and T-cells or CAR-T cells, these cells can also be considered as carriers for the virus. Injecting T-cells and human HER2-CAR-T cells with a double deleted vaccinia, vvDD-GFP to mouse showed that combining T-cell and OVV therapies causes the successful and precise delivery of the OVV to the tumor site without decreasing the individual effect of each therapy (129). Additionally, these T-cell carriers do not face the same limitations as other cell carriers, such as the elimination by the immune system. Because engineered T-cells recognize surface-expressed tumor antigens independent of antigen processing or MHC presentation, using CARs avoids the restrictions faced by TCR-activated T-cells (130).

## Recent clinical trial results of different OVVs

In recent years, many different OVVs have shown promising results in clinical trials, such as JX-929, GL-ONC1, JX-594, and others (**Table 1**). JX929 is based on the Western Reserve strain of vaccinia virus and has been engineered to selectively replicate in tumor cells by modifying the VGK and TK genes (16). This OVV has been tested first for intratumoral injection and then for intravenous injection in humans against different types of solid tumors for phase I clinical trials (16, 17). Results have shown that this OV can be administered safely, no dose limiting toxicities was observed and the virus is replicating selectively in tumor tissue (16, 17). However, clinical benefits from the administration of this virus cannot be confirmed at this moment, further experiments need to be done. The GL-ONC1 is a Lister strain OVV, it incorporated multiple gene modification such as the TK and the hemagglutinin genes (20). This OVV has been tested for intraperitoneal and intravenous injection either alone or in combination with other treatments. When tested alone or in combination with chemotherapy or with radiation, no dose limiting toxicities and little adverse effects were observed (20, 29, 32). Currently into phase III clinical trial, preliminary results of GL-ONC1 in combination with chemotherapy suggest anti-tumor activity following the infection of tumor cells, but the increased efficiency cannot be confirmed to be caused by the OV, further analysis needed to be done to validate the impact of GL-ONC1 (29). The double recombinant VV “VV-GMCSF-Lact”, which is a Lister strain based VV, is currently into phase I of clinical trial. This OVV is expressing lactaptin protein and human granulocyte-macrophage colony-stimulating factor (GM-CSF). Preliminary results indicate that this OV is a potential treatment against chemo resistant tumors (27). There is also the recombinant human IL-21 oncolytic vaccinia virus (hV01) with deletion of TK and VGF gene that is currently into phase I clinical trial. Preliminary results shown potential benefits of intratumoral expression IL-21 on TME (34). Finally, one of the most tested OVV is JX-594 also known as PexaVec. It is based on Wyeth vaccine strain of VV, it has been engineered to express GM-CSF and to selectively replicate in tumor cells by deletion of TK gene. This OV has been tested against many types of solid tumors with either intratumoral injection or intravenous infusion and as a monotherapy or in combination with other treatments (11, 12, 18, 23–26, 28, 30, 31). As monotherapy, there is a multiple clinical trial, either in phase I or II. Results suggest a potential antitumor activity offered by the OV in addition to a safe administration into the patient, the administration was well tolerated (11, 12, 23, 24, 30, 31). Few groups are now testing this OV in combination with agents like irinotecan, Durvalumab and Tremelimumab (28, 131). The first one is a chemotherapy agents and the other two are antibodies that target different immune checkpoint modulator interactions. To this date, the combination of PexaVec with immune checkpoint therapy indicates that there is no additional toxicity observed and seems to be well tolerated. In addition, the combination of treatments shows evidence that it can cause immune changes in the TME (26, 131). In general, the different clinical trials that have been done or are ongoing at this moment show that there is a lot of work to do to validate the benefits of the OVV and to optimize these treatments in order to increase the long-lasting influence and their efficacy as well as their safety. Even if there is no dose limiting toxicities observed, a number of low adverse effects have been reported, it would be interesting to see if these OVV can be optimized in order to get lower adverse effects.

## Concluding remarks and perspectives

Currently, oncolytic viruses are a promising area of cancer immunotherapy research. Gene editing technologies allow researchers to modify viruses such that they are only capable of replicating in cancerous cells while sparing healthy cells. These viruses can release tumor antigens to stimulate the patient’s immune response and simultaneously deliver a therapeutic payload. While oncolytic viruses exhibit promising anticancer potential, certain limitations exist when employing them as monotherapy. One notable constraint is the variability in treatment response among patients, influenced by the diverse immunological profiles and genetic characteristics of individual tumors (132). Moreover, tumor cells may develop resistance to oncolytic viruses over time, compromising the sustained efficacy of monotherapeutic approaches. Acknowledging the dual role of tumor vasculature, some strategies aim to collapse it for anti-cancer effects, while others focus on normalizing it for enhanced OV dissemination (6). Additionally, the host immune system may mount a robust response against the oncolytic virus, leading to its clearance before achieving optimal antitumor effects. Addressing these inherent challenges faced by oncolytic virus monotherapies thus becomes imperative in creating successful treatment strategies for patient. Combining OVs with traditional therapies such as chemotherapy or radiation can create synergistic effects, leveraging the strengths of each approach to overcome individual limitations. Furthermore, the combination of oncolytic viruses with immunomodulators, including immune checkpoint inhibitors, can augment the immune response against cancer cells, fostering a more sustained and potent antitumor effect. These combinational strategies offer a comprehensive and personalized approach to cancer treatment with improved efficacy and broader applicability. Combination OV treatments have yet to be approved but various are undergoing clinical and pre-clinical trials, with the aim of increasing response rates via additivity or synergy between treatments. This review has summarized the main oncolytic vaccinia virus combinations currently under investigation and potential explanations for this synergy. Continuing research is required to elucidate the mechanisms underlying the efficacy of these treatments to allow for their approval. Additionally, research may be expanded to study the effects of OVV with other secondary treatments including different types of chemotherapy drugs and immune therapies such as cytokines. Further research on delivery methods must also be undertaken to allow for the greatest response rates while minimizing off-target side-effects and toxicity, as well as investigating OVs for other types of cancers. The promising combination studies provide necessary evidence for continuing OV research in the hopes of having high response rates and becoming a more mainstream treatment option for patients.

In conclusion, the field of oncolytic virus therapy for cancer treatment is rapidly advancing, and combination treatments hold great promise for improving response rates and overall patient outcomes. The use of gene editing technologies has enabled the development of viruses that can specifically target cancer cells, thus avoiding harm to healthy cells. This review has highlighted the current state of oncolytic vaccinia virus combination treatments under investigation, and potential mechanisms behind the observed synergy.

Despite the encouraging results, further research is required to demonstrate the efficacy of these treatments and secure their approval for clinical use. Studies on the combination of OVs with other secondary treatments, such as chemotherapy drugs and immune therapies, should also be undertaken to further optimize treatment outcomes. Additionally, the delivery methods of oncolytic viruses must be optimized to maximize response rates while minimizing off-target side-effects and toxicity.

The future of oncolytic virus immunotherapy for cancer treatment is bright, and it holds the potential to become a mainstream treatment option for patients. As the field continues to evolve, we can expect new discoveries and advancements that will further increase response rates and improve patient outcomes. Further research and investment in this area will be crucial to realizing the full potential of oncolytic virus therapy for cancer treatment.

In light of the above, it can be concluded that the oncolytic virus therapy holds great promise as a cancer treatment option, and the combination treatments offer a particularly promising avenue for future research. The next steps should be focused on demonstrating the efficacy of these treatments and optimizing delivery methods to bring these treatments to the forefront of cancer care.

## Author contributions

TA: Conceptualization, Supervision, Writing – original draft, Writing – review & editing. SM: Conceptualization, Formal Analysis, Writing – original draft, Writing – review & editing. MD: Conceptualization, Writing – original draft, Writing – review & editing. AZ: Writing – original draft, Writing – review & editing. NK: Conceptualization, Writing – original draft, Writing – review & editing. GS: Writing – original draft, Writing – review & editing. MT: Conceptualization, Writing – original draft, Writing – review & editing.
